# The Association between Parameters of Malnutrition and Diagnostic Measures of Sarcopenia in Geriatric Outpatients

**DOI:** 10.1371/journal.pone.0135933

**Published:** 2015-08-18

**Authors:** Esmee M. Reijnierse, Marijke C. Trappenburg, Morena J. Leter, Gerard Jan Blauw, Marian A. E. de van der Schueren, Carel G. M. Meskers, Andrea B. Maier

**Affiliations:** 1 Department of Internal Medicine, Section of Gerontology and Geriatrics, VU University Medical Center, Amsterdam, The Netherlands; 2 Department of Internal Medicine, Amstelland Hospital, Amstelveen, The Netherlands; 3 Department of Gerontology and Geriatrics, Leiden University Medical Centre, Leiden, The Netherlands; 4 Department of Geriatrics, Bronovo Hospital, The Hague, The Netherlands; 5 Department of Internal Medicine, Section Nutrition and Dietetics, VU University Medical Center, Amsterdam, The Netherlands; 6 Department of Nutrition, Sports and Health, Faculty of Health and Social Studies, HAN University of Applied Sciences, Nijmegen, The Netherlands; 7 Department of Rehabilitation Medicine, VU University Medical Center, Amsterdam, The Netherlands; Ehime University Graduate School of Medicine, JAPAN

## Abstract

**Objectives:**

Diagnostic criteria for sarcopenia include measures of muscle mass, muscle strength and physical performance. Consensus on the definition of sarcopenia has not been reached yet. To improve insight into the most clinically valid definition of sarcopenia, this study aimed to compare the association between parameters of malnutrition, as a risk factor in sarcopenia, and diagnostic measures of sarcopenia in geriatric outpatients.

**Material and Methods:**

This study is based on data from a cross-sectional study conducted in a geriatric outpatient clinic including 185 geriatric outpatients (mean age 82 years). Parameters of malnutrition included risk of malnutrition (assessed by the Short Nutritional Assessment Questionnaire), loss of appetite, unintentional weight loss and underweight (body mass index <22 kg/m^2^). Diagnostic measures of sarcopenia included relative muscle mass (lean mass and appendicular lean mass [ALM] as percentages), absolute muscle mass (total lean mass and ALM/height^2^), handgrip strength and walking speed. All diagnostic measures of sarcopenia were standardized. Associations between parameters of malnutrition (independent variables) and diagnostic measures of sarcopenia (dependent variables) were analysed using multivariate linear regression models adjusted for age, body mass, fat mass and height in separate models.

**Results:**

None of the parameters of malnutrition was consistently associated with diagnostic measures of sarcopenia. The strongest associations were found for both relative and absolute muscle mass; less stronger associations were found for muscle strength and physical performance. Underweight (*p* = <0.001) and unintentional weight loss (*p* = 0.031) were most strongly associated with higher lean mass percentage after adjusting for age. Loss of appetite (*p* = 0.003) and underweight (*p* = 0.021) were most strongly associated with lower total lean mass after adjusting for age and fat mass.

**Conclusion:**

Parameters of malnutrition relate differently to diagnostic measures of sarcopenia in geriatric outpatients. The association between parameters of malnutrition and diagnostic measures of sarcopenia was strongest for both relative and absolute muscle mass, while less strong associations were found with muscle strength and physical performance.

## Introduction

Sarcopenia is a frequent syndrome in older persons, leading to functional limitations and metabolic dysregulation [1, 2]. Its clinical relevance is increasingly being recognized. The term sarcopenia literally means the ‘deficiency’ (penia) of ‘flesh’ (sarx) and was introduced in 1989 by Rosenberg [3]. Since then, several diagnostic criteria have been proposed [4]. These criteria consist of different combinations of measures of relative and absolute muscle mass, muscle strength and physical performance and different cut-off points with respect to different reference populations. Consensus working groups proposed to define sarcopenia as a syndrome characterized by the age-related loss of muscle mass and loss of muscle function, including muscle mass and strength and/or physical performance [5–10]. However, this consensus is still under debate.

Relating different measures of sarcopenia with different clinical syndromes is required to establish the most clinically valid definition. The definition of sarcopenia should be based on those diagnostic measures of sarcopenia that associate most strongly with muscle-related outcomes. In previous studies, a stronger association was reported between relative muscle mass and physical performance and glucose regulation, compared to absolute muscle mass, muscle strength and physical performance [11, 12]. In contrast, a stronger association was reported between absolute muscle mass and bone mineral density [13]. Another clinically relevant syndrome is body protein stock in relation to nutritional status [14]. Malnutrition is prevalent in approximately 20 percent of the geriatric outpatients, while more than half of these outpatients are at risk of malnutrition [15]. Malnourished older persons have an increased risk of sarcopenia due to reduced muscle protein synthesis [14]. In addition, low dietary protein intake is associated with a higher loss of lean mass in community-dwelling older persons [16]. Malnutrition and sarcopenia are both of important clinical relevance and interrelated in their pathophysiology. Exploring how parameters of malnutrition relate to diagnostic measures of sarcopenia adds insight in the most valid clinical definition of sarcopenia.

This study aimed to compare the association between parameters of malnutrition, comprising risk of malnutrition, loss of appetite, unintentional weight loss and underweight, and different diagnostic measures of sarcopenia (relative and absolute muscle mass, muscle strength and physical performance) in a clinically relevant geriatric outpatient population. Presence of an association between parameters of malnutrition and either higher relative or lower absolute muscle mass was hypothesized.

## Methods

### Study design

This cross-sectional study included 185 community dwelling older persons who were referred to a geriatric outpatient clinic in a middle sized teaching hospital (Bronovo Hospital, The Hague, The Netherlands) for a Comprehensive Geriatric Assessment (CGA) between March 2011 and January 2012. The CGA was performed during a two-hour visit including questionnaires and physical and cognitive measurements by trained nurses and medical staff. No exclusion criteria were applied; inclusion was based on the referral. Of the 185 outpatients, data on bioelectrical impedance analysis (BIA) was available in 135 consecutive outpatients due to a protocol amendment in which the BIA was added in a later stage. Data of 11 outpatients were excluded due to invalid values on measures of muscle mass, leaving 124 outpatients for the present analysis. The study was reviewed and approved by the institutional review board (IRB) of the Leiden University Medical Center (Leiden, the Netherlands). Because this research is based on regular care, the need for individual informed consent was waived by the aforementioned IRB. Ethical guidelines were followed in accordance with the Declaration of Helsinki.

### Geriatric outpatient characteristics

Anthropometric measurements were performed to assess standing height to the nearest 0.1 cm, body mass to the nearest 0.1 kg and body mass index (BMI) in kg/m^2^. Questionnaires included information about lifestyle factors such as marital status, living status, education and current smoking. Physical functioning was assessed with the Short Physical Performance Battery (SPPB) comprising balance tests with the aim to be able to maintain standing balance for ten seconds in three different positions, a timed four meter walk and a timed chair stand test [17]. A score ranging from zero to four was assigned for each of the three physical measures and a composite score ranging from zero to twelve was calculated by adding the three sub-scores. A sub-score of zero indicated that a geriatric outpatient was unable to perform the test. A total SPPB score of ≤ 10 indicated physical disability [18]. The Mini Mental State Examination (MMSE) was used to assess cognitive function [19]. The Hospital Anxiety and Depression Scale (HADS) was used to detect depressive symptoms [20]. A score higher than 8 out of 21 points indicated depressive symptoms. Comorbidity was defined as the presence of two or more chronic diseases composed of hypertension, myocardial infarction, COPD, cancer, diabetes mellitus, rheumatoid arthritis, osteoarthritis, Parkinson’s disease.

### Parameters of malnutrition

Parameters of malnutrition comprised the risk of malnutrition based on the composite score of the Short Nutritional Assessment Questionnaire (SNAQ), as well as the individual SNAQ questions on loss of appetite and unintentional weight loss [21]. In addition, a BMI of <22 kg/m^2^was regarded as parameter of malnutrition [22]. The SNAQ is a screening tool to detect patients at risk of malnutrition which includes three questions on loss of appetite, unintentional weight loss and the use of sip or tube feeding [21].

Because of the uneven distribution of cases in the subgroups of the parameters of malnutrition, these parameters were dichotomized. Outpatients were dichotomized into a group of low risk of malnutrition (composite SNAQ score <2) and medium/high risk of malnutrition (composite SNAQ score ≥2). Loss of appetite was defined as the presence of loss of appetite in the last month [21] and was dichotomized into a group of loss of appetite and a group of no loss of appetite. SNAQ defines unintentional weight loss as a loss of more than three kilograms in the previous month (representing approximately 5% weight loss) or more than six kilograms (approximately 10% weight loss) in the previous six months [21], and was dichotomized into a group of unintentional weight loss (>3 kg) and a group of no unintentional weight loss (≤3 kg). The individual SNAQ question about sip or tube feeding was included in the calculation of the SNAQ composite score [21], but excluded as a single parameter for malnutrition from statistical analysis because of the low number of cases (n = 9). Next to the individual SNAQ questions, BMI was used to define underweight using a cut-off value of <22 kg/m^2^, which is a commonly used cut-off value in the older persons population [22]. Underweight was dichotomized into a group of underweight (BMI <22 kg/m^2^) and a group of no underweight (BMI ≥22 kg/m^2^).

### Diagnostic measures of sarcopenia

#### Muscle mass

Body composition was determined using a direct segmental multi-frequency bioelectrical impedance analysis (DSM-BIA; In-Body 720; Biospace Co., Ltd, Seoul, Korea). The DSM-BIA has been shown to be a valid tool to asses whole body composition and segmental lean mass measurements with excellent agreements between DSM-BIA and dual energy X-ray absorptiometry (DXA) [23].

A distinction was made between relative and absolute muscle mass. Relative muscle mass was defined as lean mass percentage (lean mass divided by body mass) [24] and appendicular lean mass percentage (ALM, total lean mass of both arms and legs divided by body mass) [25]. Absolute muscle mass was defined as total lean mass in kilograms and ALM/height^2^ [26].

#### Muscle strength

Handgrip strength was measured to estimate muscle strength and was performed with a hand dynamometer (Jamar hand dynamometer; Sammons Preston, Inc., Bolingbrook, IL). Participants had to hold the dynamometer in their hand with the arm stretched parallel to the body and with the instruction to stand upright. This measure was performed three times on each hand and the best performance was used as the maximum handgrip strength in kilograms.

#### Physical performance

Walking speed was measured over a four-meter distance from a standing start. Participants were instructed to walk at normal pace to the end of corridor to prevent slowing down before the four meter line. A stopwatch was used in order to record the time. Walking speed was expressed in meters per second (m/s).

### Statistical analysis

Continuous variables with a normal distribution were presented as mean and standard deviation (SD). If a skewed distribution (non-Gaussian) was found, median and interquartile range (IQR) were presented.

Diagnostic measures of sarcopenia were standardized into gender-specific z-scores, to allow direct comparison of effect sizes of parameters of malnutrition with diagnostic measures of sarcopenia. Gender-specific z-scores could be calculated back to absolute values by the equation βxSD. Associations between parameters of malnutrition (independent variables) and diagnostic measures of sarcopenia (dependent variables) were analysed using multivariate linear regression models. In total, three adjustment models were constructed. In model 1, adjustment was performed for age. In model 2, further adjustments were performed for body mass or fat mass. Measures of relative muscle mass, lean mass percentage and ALM percentage, were adjusted for body mass because lower body mass is associated with malnutrition and with higher relative muscle mass. Adjustments for body mass are necessary because the fat mass/lean mass ratio changes with body mass. Handgrip strength was adjusted for body mass because a lower body mass is associated with lower handgrip strength [27]. Measures of absolute muscle mass, total lean mass and ALM/height^2^, were adjusted for fat mass since aforementioned measures do not take fat mass into account. Adjustments for fat mass are necessary because the association between absolute muscle mass and muscle-related outcomes may be influenced by fat mass [28, 29]. In model 3, further adjustments were made for height in analyses using handgrip strength and walking speed. A taller stature is associated with higher handgrip strength [27] and with faster walking speed [30]. Taller individuals have longer arms to generate more power which contributes to higher values of handgrip strength. In addition, taller individuals have longer legs to create bigger steps which contributes to a faster walking speed.

Results of the linear regression analysis with standardized variables can be interpreted as follows: the effect size (βxSD) gives the difference between outpatients with the presence of the parameter of malnutrition (risk of malnutrition) of the diagnostic measure of sarcopenia, compared to outpatients without the presence of the parameter (no risk of malnutrition). To determine the strongest association of different diagnostic measures of sarcopenia with parameters of malnutrition, effect sizes (beta) were compared.

For visualization purposes, linear regression models were used to calculate adjusted means and standard errors of the means. Visualization was performed using GraphPad Prism (version 5). Statistical analyses were performed using the Statistical Package for the Social Sciences 20.0 (SPSS Inc, Chicago, Illinois, USA). P-values below 0.05 were considered statistically significant.

## Results

### Participant characteristics

In total, 185 geriatric outpatients with a mean age of 82 years (SD 7.3) were included in this study. Characteristics of the outpatients are shown in Table 1. According to the total score of the SPPB (score ≤ 10), 149 (83%) outpatients were physically disabled. Comorbidity was present in 42% of the outpatients. Mean BMI was 25.7 kg/m^2^ (SD 4.4). In total, 29 (16%) geriatric outpatients had a risk of malnutrition based on the composite score of the SNAQ (score ≥2). In the group of 124 outpatients who underwent BIA measurements, 20 (16.1%) had a risk of malnutrition. Females reported loss of appetite more frequently than men (n = 37, 33% versus n = 14, 19%) and a higher prevalence of underweight was measured in females compared to males (n = 24, 24% versus n = 12, 17%). Weight loss was present in 24 (13%) of the geriatric outpatients.

**Table 1:** Participant characteristics, total and stratified by gender.

	N	All	Male	Female
		n = 185	n = 74	n = 111
**Socio demographics**				
Age, years	185	82.0 (7.3)	80.6 (6.9)	83.0 (7.4)
Widowed, n (%)	183	78 (42.6)	18 (24.3)	60 (55.0)
Highly educated, n (%)	184	28 (15.2)	24 (32.4)	4 (3.6)
Current smoking, n (%)	137	21 (15.3)	8 (14.3)	13 (16.0)
*Living status*, *n (%)*	184			
Nursing home		10 (5.4)	1 (1.4)	9 (8.1)
Assisted home		15 (8.2)	6 (8.2)	9 (8.1)
Home		159 (86.4)	66 (90.4)	93 (83.8)
**Health characteristics**				
SPPB score, median [IQR]	179	7 [5–10]	8 [6–10	7 [4–10]
MMSE score, median [IQR]	179	27 [24–29]	27 [24–29]	27 [24–29]
Depressive symptoms, n (%)^a^	115	30 (26.1)	17 (34.0)	13 (20.0)
Comorbidity, n (%)^b^	108	45 (41.7)	20 (40.8)	25 (42.4)
**Anthropometry**				
Body weight, kg	173	71.9 (15.6)	79.3 (12.1)	66.8 (15.1)
Height, cm	177	167.1 (9.9)	176.0 (7.1)	161.2 (6.4)
BMI, kg/m^2^	171	25.7 (4.4)	25.6 (3.6)	25.7 (5.0)
**Body composition**				
Fat mass, %	124	31.9 (9.2)	26.3 (7.0)	36.0 (8.4)
Total fat mass, kg	124	23.4 (10.2)	20.8 (7.3)	25.3 (11.5)
**Parameters of malnutrition**				
*SNAQ composite score*, *n (%)*	185			
Medium/high risk; score ≥2, n (%)		29 (15.7)	12 (16.2)	17 (15.3)
Low risk; score <2, n (%)		156 (84.3)	62 (83.8)	94 (84.7)
*SNAQ loss of appetite*, *n (%)*	185			
Loss of appetite, n (%)		51 (27.6)	14 (18.9)	37 (33.3)
No loss of appetite, n (%)		134 (72.4)	60 (81.1)	74 (66.7)
*SNAQ unintentional weight loss*, *n (%)*	185			
Unintentional weight loss; >3 kg, n (%)		24 (13.0)	10 (13.5)	14 (12.6)
No unintentional weight loss; ≤3 kg, n (%)		161 (87.0)	64 (86.5)	97 (87.4)
*SNAQ sip or tube feeding*, *n (%)*	185	9 (4.9)	3 (4.1)	6 (5.4)
*Underweight*	171			
Underweight; BMI <22 kg/m^2^, n (%)		36 (21.1)	12 (17.1)	24 (23.8)
No underweight; BMI ≥22 kg/m^2^, n (%)		135(78.9)	58 (82.9)	77 (76.2)
**Diagnostic measures of sarcopenia**				
Lean mass, %	124	64.0 (8.8)	69.4 (6.7)	60.0 (8.0)
Total lean mass, kg	124	45.7 (9.8)	53.6 (7.3)	39.9 (7.0)
ALM, %	124	28.3 (5.0)	31.4 (3.6)	26.0 (4.6)
ALM/height squared, kg/m^2^	124	7.1 (1.2)	7.8 (0.8)	6.6 (1.2)
Handgrip strength, kg	181	26.1 (8.4)	33.9 (6.1)	20.8 (5.0)
Walking speed, m/s	157	0.75 (0.29)	0.80 (0.32)	0.71 (0.26)

All variables are presented as mean (SD) unless indicated otherwise.

*SPPB* Short Physical Performance Battery, *IQR* interquartile range, *MMSE* Mini-Mental State Examination, *BMI* body mass index, *SNAQ* Short Nutritional Assessment Questionnaire, *ALM* appendicular lean mass

^a^ Depressive symptoms: Hospital Anxiety and Depression scale (HADS) score >8

^b^ Comorbidity: ≥2 chronic diseases

### Parameters of malnutrition and diagnostic measures of sarcopenia

Table 2 shows the results of the multivariate linear regression models between parameters of malnutrition and the standardized diagnostic measures of sarcopenia. Beta coefficients provide the difference of the diagnostic measure of sarcopenia between outpatients with the presence of the parameter of malnutrition and outpatients without the presence of the parameter of malnutrition. For the interpretation of the results, beta coefficients were used to calculate the absolute effect size (βxSD) of the diagnostic measures for sarcopenia in percentage, kilograms or meters per second.

**Table 2:** Associations between parameters of malnutrition and standardized gender-specific diagnostic measures of sarcopenia.

	Risk of malnutrition			Loss of appetite			Unintentional weight loss			Underweight		
	β	SE	*p*	β	SE	*p*	β	SE	*p*	β	SE	*p*
**Relative muscle mass**												
*Z Lean mass percentage (%)* ^a^												
Model 1 (age)	0.34	0.24	0.161	–0.38	0.19	0.050	0.57	0.26	**0.031**	1.01	0.20	**<0.001**
Model 2 (as 1 and body mass)	0.13	0.22	0.544	–0.56	0.17	**0.001**	0.33	0.24	0.166	NA		
*Z ALM percentage (%)* ^a^												
Model 1 (age)	0.17	0.24	0.475	–0.19	0.19	0.320	0.28	0.26	0.285	0.84	0.20	**<0.001**
Model 2 (as 1 and body mass)	0.06	0.24	0.796	–0.29	0.19	0.130	0.15	0.26	0.558	NA		
**Absolute muscle mass**												
*Z Total lean mass (kg)* ^a^												
Model 1 (age)	–0.37	0.24	0.124	–0.49	0.19	**0.011**	–0.29	0.26	0.273	–0.82	0.21	**<0.001**
Model 2 (as 1 and fat mass)	–0.21	0.23	0.350	–0.54	0.18	**0.003**	–0.07	0.25	0.773	–0.52	0.22	**0.021**
*Z ALM/height* ^*2*^ *(kg/m* ^*2*^ *)* ^a^												
Model 1 (age)	–0.52	0.24	**0.032**	–0.34	0.20	0.087	–0.47	0.27	0.078	–0.89	0.21	**<0.001**
Model 2 (as 1 and fat mass)	–0.36	0.23	0.112	–0.37	0.18	**0.036**	–0.26	0.25	0.312	–0.61	0.23	**0.008**
**Muscle strength**												
*Z Handgrip strength (kg)* ^b^												
Model 1 (age)	–0.32	0.20	0.113	–0.43	0.16	**0.007**	–0.39	0.22	0.076	–0.18^d^	0.18	0.312
Model 2 (as 1 and body mass)	–0.28	0.20	0.156	–0.36	0.16	**0.026**	–0.37	0.22	0.088	NA		
Model 3 (as 2 and height)	–0.37	0.20	0.067	–0.31	0.16	0.051	–0.44	0.22	**0.040**	NA		
**Physical performance**												
*Z Walking speed (m/s)* ^c^												
Model 1 (age)	–0.35	0.24	0.151	–0.40	0.18	**0.031**	–0.34	0.26	0.194	0.06^e^	0.21	0.769
Model 2 (as 1 and height)	–0.35	0.25	0.157	–0.37	0.19	0.052	–0.35	0.26	0.172	NA		

β beta, *SE* standard error, *p* p-value, *ALM* appendicular lean mass, *NA* not applicable.

All diagnostic measures of sarcopenia were standardized and presented in gender-specific z-scores.

Interpretation: The β gives the difference between outpatients with the presence of the parameter of malnutrition of the diagnostic measure of sarcopenia in SD, compared to outpatients without the presence of the parameter. E.g.: Outpatients with risk of malnutrition have 0.40 higher z-score lean mass percentage, compared to outpatients with no risk of malnutrition.

*P*-values in bold are statistically significant

Data available in a subgroup of ^a^ n = 123

^b^ n = 180

^c^ n = 156

^d^ n = 168

^e^ n = 148

#### Relative muscle mass

There was no statistically significant association between risk of malnutrition and lean mass percentage. Loss of appetite was associated with a lower lean mass percentage of 4.9% after adjusting for age and body mass. Unintentional weight loss was associated with a higher lean mass percentage of 5.0% after adjusting for age, but this association disappeared after adjusting for body mass. Underweight was associated with a higher lean mass percentage of 8.9% after adjusting for age.

There were no statistically significant associations between risk of malnutrition, loss of appetite, unintentional weight loss, and ALM percentage. Underweight was associated with a higher ALM percentage of 4.2% after adjusting for age.

#### Absolute muscle mass

There was no association between risk of malnutrition and total lean mass. Loss of appetite was associated with a lower total lean mass of 4.8 kg after adjusting for age. This association became stronger after adjusting for fat mass (5.3 kg). Unintentional weight loss was not associated with total lean mass. Underweight was associated with a lower total lean mass of 8.0 kg after adjusting for age. This association attenuated after adjusting for fat mass (5.1 kg).

Risk of malnutrition was associated with a lower ALM/height^2^ of 0.6 kg/m^2^ after adjusting for age, but this association disappeared after adjusting for fat mass. Loss of appetite was associated with a lower ALM/height^2^ of 0.5 kg/m^2^ after adjusting for age and fat mass. Underweight was associated with a lower ALM/height^2^ of 1.1 kg/m^2^ after adjusting for age. This association attenuated after adjusting for fat mass (0.7 kg/m^2^).

#### Muscle strength

There was no statistically significant association between risk of malnutrition and handgrip strength. Loss of appetite was associated with a lower handgrip strength of 3.6 kg after adjusting for age. This association attenuated after adjusting for body mass (3.0 kg) and disappeared after adjusting for height. Unintentional weight loss was associated with a lower handgrip strength of 3.7 kg after adjusting for age, body mass and height. Underweight was not statistically significant associated with handgrip strength.

#### Physical performance

Risk of malnutrition was not statistically significant associated with walking speed. Loss of appetite was associated with a lower walking speed of 0.12 m/s after adjusting for age. This association disappeared after adjusting for age. Unintentional weight loss and underweight were not associated with walking speed.

### Comparison of diagnostic measures of sarcopenia

Comparing the effect sizes (beta), none of the parameters of malnutrition appeared to be consistently associated with diagnostic measures of sarcopenia. The strongest associations were found for both relative and absolute muscle mass. Unintentional weight loss and underweight were most strongly associated with higher relative muscle mass. Loss of appetite and underweight were most strongly associated with lower absolute muscle mass.

**Fig 1:**
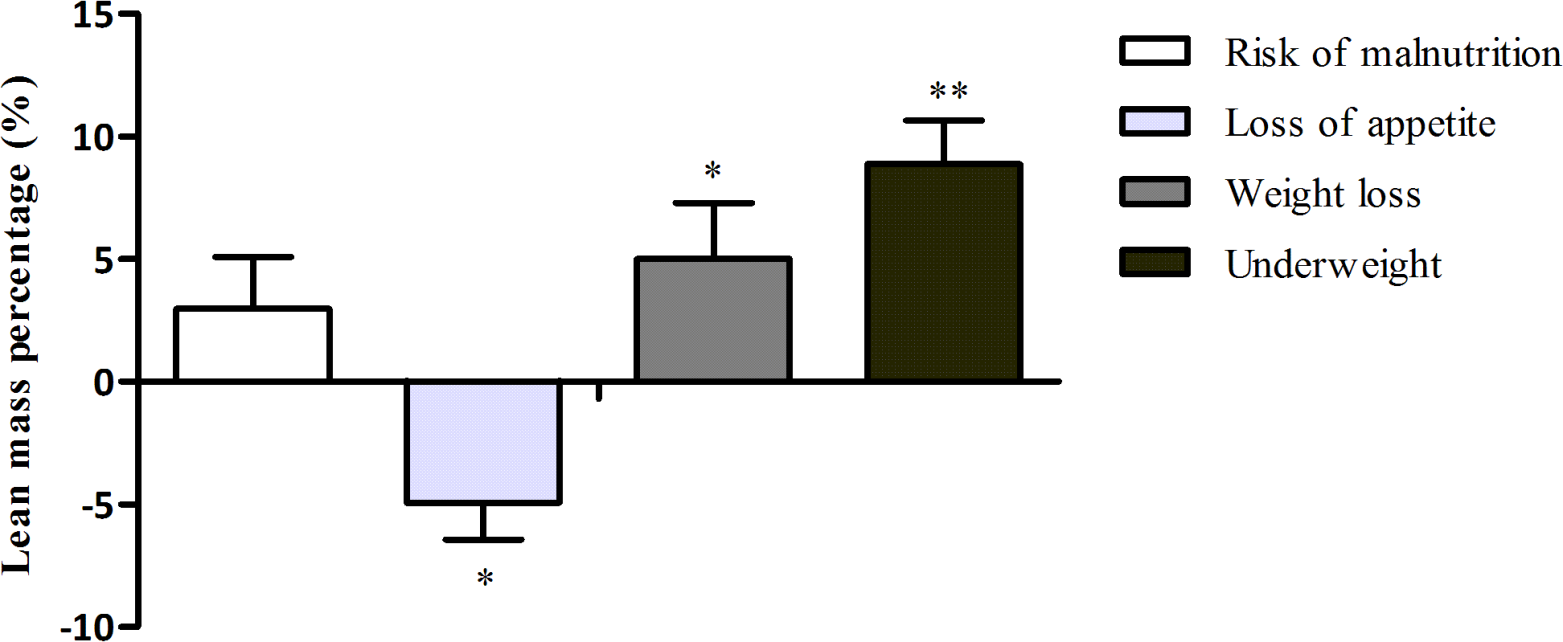
The association between parameters of malnutrition and relative muscle mass, expressed as lean mass percentage. Lean mass as percentage of body mass is presented as mean (SD) of gender-specific z-scores. Bars represent the difference of lean mass percentage between older persons with the presence of the parameter of malnutrition compared to older persons without the presence of the parameters. P values were calculated with multivariate linear regression analysis including adjustments for age (and body mass in the association with loss of appetite). * = p<0.05. ** = p<0.001.

**Fig 2:**
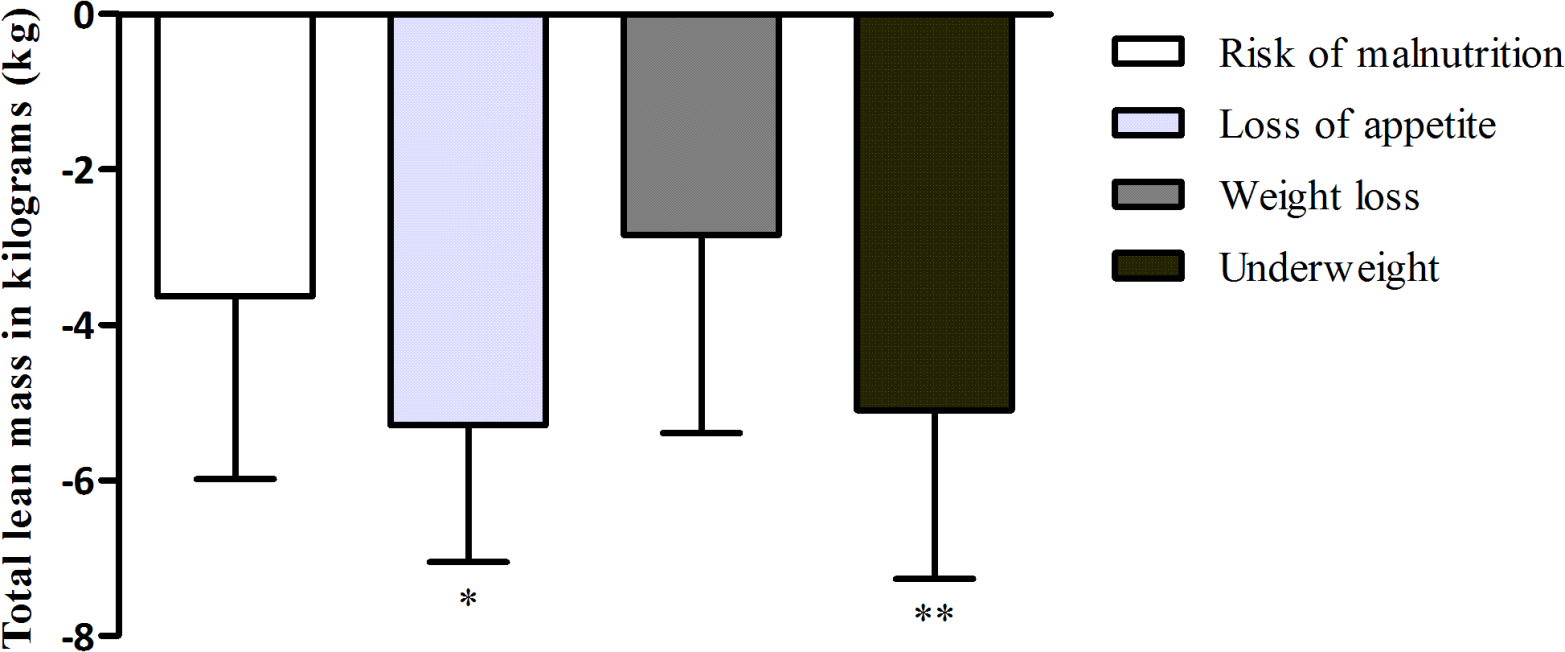
The association between parameters of malnutrition and absolute muscle mass, expressed as total lean mass. Total lean mass is presented as mean (SD) of gender-specific z-scores. Bars represent the difference of total lean mass between older persons with the presence of the parameter of malnutrition compared to older persons without the presence of the parameters. P values were calculated with multivariate linear regression analysis including adjustments for age (and fat mass and height in the association with loss of appetite and underweight). * = p<0.05. ** = p<0.001.

Figs 1 and 2 show the association between parameters of malnutrition and relative muscle mass, expressed as lean mass percentage and absolute muscle mass, expressed as total lean mass.

## Discussion

The purpose of this study was to compare the associations of parameters of malnutrition with diagnostic measures of sarcopenia in geriatric outpatients. Parameters of malnutrition did not consistently associate with diagnostic measures of sarcopenia, which indicates that measures cannot be used interchangeably. The association of parameters of malnutrition with diagnostic measures of sarcopenia was reflected by both relative and absolute muscle mass; less strong associations were found between parameters of malnutrition and muscle strength and physical performance. Unintentional weight loss and underweight were most strongly associated with higher relative muscle mass. Loss of appetite and underweight were most strongly associated with lower absolute muscle mass.

### Muscle mass

In geriatric outpatients, unintentional weight loss was associated with a higher lean mass percentage. In the prospective Health, Aging, and Body Composition Study carried out in a general population of older persons, weight loss was associated with relative loss of lean mass over a time period of four years [31]. However, the aforementioned study is not entirely comparable with the current study because of its longitudinal design in which weight loss was measured over a four-year period. In the current study, outpatients with underweight had a higher lean mass percentage of 8.9%. Comparably, in a cross-sectional study carried out in older persons from a geriatric hospital, lean mass percentage was significantly higher in underweight older persons (males 5.1%, females 10.4%) compared to older persons with a normal weight [32]. However, a cut-off value of BMI <20 kg/m^2^ was used to define underweight [32] which is not similar to the cut-off value of BMI <22 kg/m^2^ used in the current study. It is still under debate which cut-off value of BMI should be used in older persons and studies are difficult to compare due to the use of these different cut-off values for BMI.

Loss of appetite was associated with a lower lean mass percentage, which is in contrast with the other parameters of malnutrition; unintentional weight loss and underweight that were associated with a higher lean mass percentage. Loss of appetite was also associated with a lower total lean mass. Loss of appetite is a multifactorial and subjective measure and its mechanism is still not completely understood [33]. To the best of our knowledge, these associations have not been studied before.

Underweight was associated with a lower total lean mass of 8.0 kg which supports the hypothesis that parameters of malnutrition associate with a lower amount of absolute muscle mass. This association has also been reported in a cross-sectional study carried out in older persons from a geriatric hospital. In underweight older persons, total lean mass was significantly lower (males -17.0 kg, females -7.9 kg) compared to older persons with a normal weight [32]. However, a different cut-off value was used to define underweight (BMI <20 kg/m^2^).

In outpatients with underweight, a lower amount of absolute muscle mass was found in contrast to a higher amount of relative muscle mass. The differences between relative and absolute muscle mass can be explained by the fact that in a chronic underweight condition, muscle mass is preserved relatively to fat mass. Therefore, underweight older persons may have higher relative muscle mass in relation to their body mass [28, 29]. Body composition changes significant with increasing age [34], including a decrease in absolute muscle mass [35]. In addition, acute malnutrition is associated with loss of lean mass while chronic malnutrition is associated with severe loss of fat mass. Therefore, in the presence of underweight it is important to take both relative and absolute muscle mass into account when defining sarcopenia.

### Muscle strength and physical performance

Loss of appetite and unintentional weight loss were associated with a lower handgrip strength. To the best of our knowledge, these associations with handgrip strength have not been studied before. In the current study, loss of appetite was associated with a lower walking speed of 0.12 m/s. This result is in line with a previous study carried out in community-dwelling older persons [36]. Walking speed was found to be 0.12 m/s lower in older persons with loss of appetite compared to older persons with no loss of appetite, adjusted for age, gender, BMI, number of diseases, depression, congestive heart failure and lung diseases [36]. However, the associations with muscle strength and physical performance in the current study were less stronger compared to the association of parameters of malnutrition with relative and absolute muscle mass.

### Comparison with previous studies on diagnostic measures of sarcopenia and muscle related outcomes

When comparing diagnostic measures of sarcopenia in the current study with previous studies, there are some aspects that need to be considered. First, the current study includes a geriatric outpatient population in which no exclusion criteria were applied. This is in contrast to previous studies including a healthy old population [11–13]. Second, previous studies used other adjustment models, which makes comparing these studies difficult [11–13, 37]. Therefore, the results are discussed with respect to different populations and different adjustment models.

In the current study, parameters of malnutrition were found to be most strongly associated with relative and absolute muscle mass. In healthy older persons, relative muscle mass was found to be most strongly associated with physical performance [11] and glucose regulation [12], and absolute muscle mass was most strongly associated with bone mineral density [13]. In one study in geriatric outpatients, no association was found between relative and absolute muscle mass and physical performance measured by standing balance [37]. As was found in the current study, older persons with underweight may have higher muscle mass relative to their body mass while absolute muscle mass is lower and decreases with increasing chronological age [34]. Therefore, it is important to distinguish between relative and absolute muscle mass when defining sarcopenia and to take the different populations studied into account.

### Strengths and limitations

To the best of our knowledge, this is the first study comparing parameters of malnutrition with different diagnostic measures of sarcopenia in geriatric outpatients. A geriatric outpatient population is an unique population and relevant for clinical practice. However, the results cannot be generalized to other populations and selection bias could have occurred because of this specific population. There are also some other limitations that need to be addressed. First, the use of cross-sectional data hinders determination of causality. Second, SNAQ as a proxy of malnutrition in a geriatric outpatient population has a moderate sensitivity; the specificity, however, is excellent [38]. In addition, the SNAQ is a subjective measure with a possibility of recall bias, while objective measures of malnutrition are preferred. Third, the small sample size can possibly explain the lack of consistently statistical significance. Fourth, all parameters of malnutrition were dichotomized, which may affected the power of the statistical analyses. In addition, the used cut-off value of BMI <22 kg/m^2^ to define underweight was arbitrary; the debate on proper cut-off values of BMI in older persons is on-going.

### Conclusion

Parameters of malnutrition were found to be associated with diagnostic measures of sarcopenia, but related differently to diagnostic measures of sarcopenia. When comparing different diagnostic measures of sarcopenia, parameters of malnutrition were most strongly associated with both relative and absolute muscle mass, while less strong associations were found with muscle strength and physical performance. This result adds to the evidence on the importance of the role of muscle mass in defining sarcopenia. This understanding is essential for the construction of the most clinically valid definition of sarcopenia. With respect to malnutrition, both relative and absolute muscle mass are important measures.

Further research is needed and should focus on the association between the diagnostic measures of sarcopenia and other muscle-related conditions in clinically relevant populations of older persons. Interrelations are complex, i.e. effects of age, body mass, chronic and acute malnutrition. Future research should therefore carefully take adjustment models into account and should apply adjustments in a consistent way as the influence of these factors is clearly shown.

## References

[1] Visser M, Goodpaster BH, Kritchevsky SB, Newman AB, Nevitt M, Rubin SM, et al Muscle mass, muscle strength, and muscle fat infiltration as predictors of incident mobility limitations in well-functioning older persons. J Gerontol A Biol Sci Med Sci. 2005;60(3):324–33. 10.1093/gerona/60.3.324 15860469

[2] Welch AA. Nutritional influences on age-related skeletal muscle loss. Proc Nutr Soc. 2013:1–18. 10.1017/S0029665113003698 24229650

[3] Rosenberg IH. Sarcopenia: origins and clinical relevance. J Nutr. 1997;127(5):990S–1S. 9164280 10.1093/jn/127.5.990S

[4] Bijlsma A, Meskers C, Ling C, Narici M, Kurrle S, Cameron I, et al Defining sarcopenia: the impact of different diagnostic criteria on the prevalence of sarcopenia in a large middle aged cohort. Age. 2013;35(3):871–81. 10.1007/s11357-012-9384-z 22314402 PMC3636407

[5] Cruz-Jentoft AJ, Baeyens JP, Bauer JM, Boirie Y, Cederholm T, Landi F, et al Sarcopenia: European consensus on definition and diagnosis Report of the European Working Group on Sarcopenia in Older People. Age Ageing. 2010;39(4):412–23. 10.1093/ageing/afq034 20392703 PMC2886201

[6] Fielding RA, Vellas B, Evans WJ, Bhasin S, Morley JE, Newman AB, et al Sarcopenia: an undiagnosed condition in older adults. Current consensus definition: prevalence, etiology, and consequences. International working group on sarcopenia. J Am Med Dir Assoc. 2011;12(4):249–56. 10.1016/j.jamda.2011.01.003 21527165 PMC3377163

[7] Chen L-K, Liu L-K, Woo J, Assantachai P, Auyeung T-W, Bahyah KS, et al Sarcopenia in Asia: consensus report of the Asian Working Group for Sarcopenia. J Am Med Dir Assoc. 2014;15(2):95–101. 10.1016/j.jamda.2013.11.025 24461239

[8] Morley JE, Abbatecola AM, Argiles JM, Baracos V, Bauer J, Bhasin S, et al Sarcopenia with limited mobility: an international consensus. J Am Med Dir Assoc. 2011;12(6):403–9. 10.1016/j.jamda.2011.04.014 21640657 PMC5100674

[9] Muscaritoli M, Anker S, Argiles J, Aversa Z, Bauer J, Biolo G, et al Consensus definition of sarcopenia, cachexia and pre-cachexia: joint document elaborated by Special Interest Groups (SIG)“cachexia-anorexia in chronic wasting diseases” and “nutrition in geriatrics”. Clin Nutr. 2010;29(2):154–9. 10.1016/j.clnu.2009.12.004 20060626

[10] Studenski SA, Peters KW, Alley DE, Cawthon PM, McLean RR, Harris TB, et al The FNIH Sarcopenia Project: Rationale, Study Description, Conference Recommendations, and Final Estimates. J Gerontol A Biol Sci Med Sci. 2014;69(5):547–58. 10.1093/gerona/glu010 24737557 PMC3991146

[11] Bijlsma A, Meskers C, van den Eshof N, Westendorp R, Sipilä S, Stenroth L, et al Diagnostic criteria for sarcopenia and physical performance. AGE. 2014;36(1):275–85. 10.1007/s11357-013-9556-5 23818105 PMC3889901

[12] Bijlsma A, Meskers C, van Heemst D, Westendorp R, de Craen A, Maier A. Diagnostic criteria for sarcopenia relate differently to insulin resistance. AGE. 2013:1–9. 10.1007/s11357-013-9516-0 PMC3824998 23407994

[13] Bijlsma A, Meskers M, Molendijk M, Westendorp R, Sipilä S, Stenroth L, et al Diagnostic measures for sarcopenia and bone mineral density. Osteoporos Int. 2013:1–11. 10.1007/s00198-013-2376-8 23649802

[14] Boirie Y, Morio B, Caumon E, Cano NJ. Nutrition and protein energy homeostasis in elderly. Mech Ageing Dev. 2014;136:76–84. 10.1016/j.mad.2014.01.008 24486557

[15] van Bokhorst-de van der Schueren MA, Lonterman-Monasch S, de Vries OJ, Danner SA, Kramer MH, Muller M. Prevalence and determinants for malnutrition in geriatric outpatients. Clin Nutr. 2013;32(6):1007–11. 10.1016/j.clnu.2013.05.007 23755842

[16] Houston DK, Nicklas BJ, Ding J, Harris TB, Tylavsky FA, Newman AB, et al Dietary protein intake is associated with lean mass change in older, community-dwelling adults: the Health, Aging, and Body Composition (Health ABC) Study. Am J Clin Nutr. 2008;87(1):150–5. 18175749 10.1093/ajcn/87.1.150

[17] Guralnik JM, Simonsick EM, Ferrucci L, Glynn RJ, Berkman LF, Blazer DG, et al A short physical performance battery assessing lower extremity function: association with self-reported disability and prediction of mortality and nursing home admission. J Gerontol. 1994;49(2):M85–M94. 10.1093/geronj/49.2.M85 8126356

[18] Vasunilashorn S, Coppin AK, Patel KV, Lauretani F, Ferrucci L, Bandinelli S, et al Use of the Short Physical Performance Battery Score to predict loss of ability to walk 400 meters: analysis from the InCHIANTI study. The Journals of Gerontology Series A: Biological Sciences and Medical Sciences. 2009;64(2):223–9. 10.1093/gerona/gln022 PMC2655026 19182232

[19] Folstein MF, Folstein SE, McHugh PR. “Mini-mental state”: a practical method for grading the cognitive state of patients for the clinician. J Psychiatr Res. 1975;12(3):189–98. 10.1016/0022-3956(75)90026-6 1202204

[20] Zigmond AS, Snaith RP. The hospital anxiety and depression scale. Acta Psychiatr Scand. 1983;67(6):361–70. 6880820 10.1111/j.1600-0447.1983.tb09716.x

[21] Kruizenga H, Seidell J, De Vet H, Wierdsma N, van Bokhorst–de van der Schueren M. Development and validation of a hospital screening tool for malnutrition: the short nutritional assessment questionnaire (SNAQ). Clin Nutr. 2005;24(1):75–82. 10.1016/j.clnu.2004.07.015 15681104

[22] Ferreira RS, da Silva Coqueiro R, Barbosa AR, Pinheiro PA, Fernandes MH. Relationship between BMI and physical performance among older adults. Geriatr Nurs. 2013;34(6):465–8. 10.1016/j.gerinurse.2013.07.013 23993659

[23] Ling CH, de Craen AJ, Slagboom PE, Gunn DA, Stokkel MP, Westendorp RG, et al Accuracy of direct segmental multi-frequency bioimpedance analysis in the assessment of total body and segmental body composition in middle-aged adult population. Clin Nutr. 2011;30(5):610–5. 10.1016/j.clnu.2011.04.001 21555168

[24] Janssen I, Heymsfield SB, Ross R. Low relative skeletal muscle mass (sarcopenia) in older persons is associated with functional impairment and physical disability. J Am Geriatr Soc. 2002;50(5):889–96. 10.1046/j.1532-5415.2002.50216.x 12028177

[25] Estrada M, Kleppinger A, Judge JO, Walsh SJ, Kuchel GA. Functional impact of relative versus absolute sarcopenia in healthy older women. J Am Geriatr Soc. 2007;55(11):1712–9. 10.1111/j.1532-5415.2007.01436.x 17979895

[26] Baumgartner RN, Koehler KM, Gallagher D, Romero L, Heymsfield SB, Ross RR, et al Epidemiology of sarcopenia among the elderly in New Mexico. Am J Epidemiol. 1998;147(8):755–63. 9554417 10.1093/oxfordjournals.aje.a009520

[27] Guerra RS, Fonseca I, Pichel F, Restivo MT, Amaral TF. Handgrip Strength and Associated Factors in Hospitalized Patients. J Parenter Enteral Nutr. 2013;0148607113514113. 10.1177/0148607113514113 24291737

[28] Delmonico MJ, Harris TB, Lee JS, Visser M, Nevitt M, Kritchevsky SB, et al Alternative definitions of sarcopenia, lower extremity performance, and functional impairment with aging in older men and women. J Am Geriatr Soc. 2007;55(5):769–74. 10.1111/j.1532-5415.2007.01140.x 17493199

[29] Lebrun CE, van der Schouw YT, de Jong FH, Grobbee DE, Lamberts SW. Fat mass rather than muscle strength is the major determinant of physical function and disability in postmenopausal women younger than 75 years of age. Menopause. 2006;13(3):474–81. 10.1097/01.gme.0000222331.23478.ec 16735945

[30] Tolea MI, Costa PT, Terracciano A, Griswold M, Simonsick EM, Najjar SS, et al Sex-specific correlates of walking speed in a wide age-ranged population. J Gerontol B Psychol Sci Soc Sci. 2010;65(2):174–84. 10.1093/geronb/gbp130 PMC2821942 20051464

[31] Newman AB, Lee JS, Visser M, Goodpaster BH, Kritchevsky SB, Tylavsky FA, et al Weight change and the conservation of lean mass in old age: the Health, Aging and Body Composition Study. Am J Clin Nutr. 2005;82(4):872–8. 16210719 10.1093/ajcn/82.4.872

[32] Sergi G, Coin A, Bussolotto M, Benincà P, Tomasi G, Pisent C, et al Influence of fat-free mass and functional status on resting energy expenditure in underweight elders. J Gerontol A Biol Sci Med Sci. 2002;57(5):M302–M7. 10.1093/gerona/57.5.M302 11983724

[33] Martone AM, Onder G, Vetrano DL, Ortolani E, Tosato M, Marzetti E, et al Anorexia of aging: a modifiable risk factor for frailty. Nutrients. 2013;5(10):4126–33. 10.3390/nu5104126 24128975 PMC3820063

[34] Baumgartner RN, Stauber PM, McHugh D, Koehler KM, Garry PJ. Cross-sectional age differences in body composition in persons 60+ years of age. J Gerontol A Biol Sci Med Sci. 1995;50(6):M307–M16. 10.1093/gerona/50A.6.M307 7583802

[35] Visser M, Kritchevsky SB, Goodpaster BH, Newman AB, Nevitt M, Stamm E, et al Leg muscle mass and composition in relation to lower extremity performance in men and women aged 70 to 79: the Health, Aging and Body Composition study. J Am Geriatr Soc. 2002;50(5):897–904. 10.1046/j.1532-5415.2002.50217.x 12028178

[36] Landi F, Russo A, Liperoti R, Tosato M, Barillaro C, Pahor M, et al Anorexia, physical function, and incident disability among the frail elderly population: Results from the ilSIRENTE study. J Am Med Dir Assoc. 2010;11(4):268–74. 10.1016/j.jamda.2009.12.088 20439047

[37] Bijlsma AY, Pasma JH, Lambers D, Stijntjes M, Blauw GJ, Meskers CG, et al Muscle Strength Rather Than Muscle Mass Is Associated With Standing Balance in Elderly Outpatients. J Am Med Dir Assoc. 2013;14(7):493–8. 10.1016/j.jamda.2013.02.001 23540951

[38] Schilp J, Leistra E, de Vries OJ, Visser M, Kruizenga HM. Ondervoeding herkennen in het Centrum voor Ouderengeneeskunde Amsterdam. Ned Tijdschr Evid Based Pract. 2011;9(3):18–23. Article in Dutch.

